# L-theanine: an astounding sui generis amino acid in poultry nutrition

**DOI:** 10.1016/j.psj.2020.07.016

**Published:** 2020-08-06

**Authors:** Muhammad Saeed, Muhammad Sajjad Khan, Asghar Ali Kamboh, Mahmoud Alagawany, Asmaa F. Khafaga, Ahmed E. Noreldin, Muhammad Qumar, Muhammad Safdar, Mubashar Hussain, Mohamed E. Abd El-Hack, Sun Chao

**Affiliations:** ∗ Northwest A&F University, Yangling 712100, PR China; † Faculty of Animal Production and Technology, Cholistan University of Veterinary and Animal Sciences, Bahawalpur 63100, Pakistan; ‡ Department of Veterinary Microbiology, Faculty of Animal Husbandry and Veterinary Sciences, Sindh Agriculture University, Tandojam 70060, Pakistan; § Department of Poultry, Faculty of Agriculture, Zagazig University, Zagazig 44511, Egypt; # Department of Pathology, Faculty of Veterinary Medicine, Alexandria University, Edfina 22758, Egypt; ‖‖ Department of Histology and Cytology, Faculty of Veterinary Medicine, Damanhour University, Damanhour 22516, Egypt; ∗∗ Institute of Animal and Dairy Sciences, University of Agriculture, Faisalabad, Pakistan

**Keywords:** L-theanine, tea plant, amino acid, biological effect, poultry

## Abstract

L-theanine (γ-Glutamylethylamide) is a nonprotein water soluble amino acid (**AA**) mostly found in leaves of *Camellia sinensis* (green tea). This is a key component of green tea and is considered as the most abundant form of total AAs in green tea (i.e., about 50%). L-theanine is an exclusive taste ingredient of tea producing an attractive flavor and aroma in tea. It has biological effects such as antioxidant, growth promoter, immune booster, anti-stresser, hepatoprotective, antitumor, antiaging, antimicrobial, anti-inflammatory, and antianxiety activities that are worth noticing. It could reduce the oxidative impairment by reducing the synthesis of reactive oxygen species, oxidative parameters, and lipid damage as well as increasing the activity of antioxidant enzymes. The oral ingestion of L-theanine enhanced γδ T-cell proliferation. Therefore, it is being considered an essential compound of green tea that has the ability to improve immune function. The L-theanine can be used as a potential treatment for hepatic injury and immune-related liver diseases via the downregulation of the inflammatory response through the initiation of nitric oxide synthesis and glutathione production which are likely to be critical for the control of hepatic diseases as well as for the improvement of immune function. In addition, it could be used as a best natural feed additive with a potent antistressor by decreasing the levels of corticosterone, dopamine, and noradrenaline. After systematically reviewing the literature, it is noticed that most studies were carried out on mice, pig, human, and butterfly; while dietary supplementation studies of L-theanine in animal and poultry especially among broilers are very limited because of less awareness of this AA. So, the aim of this review is to encourage the veterinarian and poultry researchers to conduct more research at the molecular level about this AA to expose its more beneficial effects and its mechanism of absorption for potential use of this unique green tea AA in poultry nutrition.

## Introduction

Tea is derived from the leaves of *Camellia sinensis* (green tea) and is one of the utmost traditionally consumed beverages throughout the globe. Historically, tea has been used as a medicinal herb dating back to 4,700 yr in China and is regarded as a healthy practice, especially for improving liver function. Although caffeine and catechins are the primary biological components considered to contribute to the beneficial effects of tea, the health benefits of theanine have also become prominent in recent years. L-theanine is a nonproteinic amino acid (**AA**) that is found in the tea plant. The International Union of Pure and Applied Chemistry proposed the name of theanine as 2-amino-4-(ethylcarbamoyl) butyric acid (Vuong et al., 2011). Similar to other natural AAs, L-theanine is a chiral type and is mainly observed in the L-enantiomer form (Wan et al., 2008). L-theanine is a unique taste element of tea with an attractive aroma and a caramel flavor that aid to attenuate the bitterness of caffeine (Eschenauer and Sweet, 2006). Toxicological and technical assessment tests proposed that L-theanine is a nontoxic and safe phytogenic food additive. L-theanine is well known with several different names including γ–glutamylethylamide and γ-ethylamino-l-glutamic acid, and it also has a commercial name called Suntheanine (Facts and Comparisons, 2007). L-theanine (γ-glutamylethylamide) is a non–protein-derived AA that is bountiful in leaves of green tea (Deng et al., 2010). It (γ-glutamylethylamide) has been evaluated as a natural food additive for food fortification in relation to human nutrition and health benefits. It has many biological and pharmacological activities such as cerebral ischemia/reperfusion injury protection, antitumor, stress modulator, antiaging, and antianxiety properties (Higashiyama et al., 2011; Saito et al., 2011; Gong et al., 2012; Culetu et al., 2016). In addition, Cooper (2012) pointed out that dietary inclusion of L. theanine is an easy way to alleviate ROS (reactive oxygen species)-induced damage.

In the tea, L-theanine is biosynthesized from ethylamine and glutamic acid via the enzyme theanine synthetase (Deng et al., 2008). It represents nearly 50% of the total AAs in leaves of tea. It covers approximately 1 to 2% of the total dry weight (**DM**) of green tea leaves with one cup of green tea containing about 8 to 30 mg of theanine (de Mejia et al., 2009). L-theanine is readily bioavailable on consumption and is quickly absorbed in the intestinal tract followed by its metabolism in the liver (Owen et al., 2008; Van der Pijl et al., 2010). Chronic and acute toxicity tests carried out on the safety of L-theanine have not so far confirmed its toxicity (Turkozu and Sanlier, 2017).

L-theanine is available as a dietary supplement and has been approved as “generally recognized as safe” by the U.S. Food and Drug Administration Authority (Wang et al., 2017). Several researches have stated that L-theanine decreases the oxidative impairment by reducing the production of ROS, oxidative parameters, and lipid damage as well as enhancing the activity of antioxidative enzymes (Ben et al., 2015). Furthermore, L-theanine improved hepatocyte antioxidant capacity by inhibiting the malondialdehyde (**MDA**) formation and increasing the antioxidant enzymes activities such as catalase (**CAT**) and superoxide dismutase (**SOD**) and reduced GSH during liver damage in *in vivo* rat model and *in vitro* studies (Li et al., 2012; Thangarajan et al., 2014). Its intraduodenal and intraruminal administration in cattle has been proved effective against liver diseases (Winkler et al., 2015). Several previous animal studies, epidemiological studies, and human interventions support the conclusion that tea and tea extracts have a promising protective effect on liver tissue (Mehana et al., 2012). For example, in the study of Ruhl and Everhart (2005), they reported that the ingestion of tea (>2 cups per day) is linked to a lower occurrence of chronic liver disease in the United States. Although some studies on tea have focused on catechins, L-theanine may be the compound that mediates the hepatoprotective capabilities of tea. Several other published reports indicated the hepatoprotective activities of L-theanine on toxicity-induced hepatic injury and its pharmacokinetic and pharmacodynamic properties. Yang et al. (2013) reported that L-theanine when fed to fruit fly resulted in a significant effect against stress via dry and wet starvation in males. Li et al. (2016) reported that supplementation of L-theanine at the rate of 400 mg/kg BW/d for 14 d in rat positively improved immune function by increasing the spleen function and decreasing the corticosterone (**CORT**) level in the serum of rat.

In this review, theanine will be referred to as specifically L-theanine. The review consisted of available scientific evidences about L-theanine pertaining to its chemical composition, antistressor and immune functions, and effects on performance in different animal models such as mice, pigs, and so on. To the best of the authors' knowledge, most of the previous studies were conducted in human, mice, and pigs, and there is a serious gap of information in literature on the use of L-theanine in farm animal and birds especially in broiler production. So, this is the first review article on L-theanine which would provide exclusively a new insight regarding this AA that could be used as a best natural feed additive with antistressor and hepatoprotective properties in poultry farming. This article compiled its natural sources, mechanism of actions, chemical composition, and beneficial applications in animal and poultry health. Moreover, this review will be useful for poultry nutritionists, veterinarians, and researchers, to expose its more beneficial effects, so it could be used as dietary supplement in poultry feed.

## Sources and structure of L-theanine

Naturally, this AA (theanine) is derived from a nonedible mushroom, *Xerocomus badius* and *Camellia* genus including *C. sinensis* var. *assamica*, *C. sinensis* var. *sinensis*, *Camellia sasanqua*, and *Camellia japonica* (Deng et al., 2008). It shares in tea aroma in a great concentration and particularly it is linked to the umami taste of the tea (Narukawa et al., 2014). L-theanine consists of 50% of the free AAs in tea. The level of L-theanine in the tea represents 1 to 3% of the dry tea, and that level varies depending on many factors such as the geographic region in which the tea is grown, conditions of cultivation, variety of tea, time and harvest type, and so on (Vuong et al., 2011). The type of tea is essential as well in terms of theanine amount, for example, comparing with *C. sinensis* var. *assamica*, *C. sinensis* var. *sinensis* have higher contents of L-theanine as confirmed by Chu (1997). In addition, the harvested tea in the early period of the summer has greater L-theanine contents in contrast to tea cultivated in the late summer season (Vuong et al., 2011).

**Figure 1:**
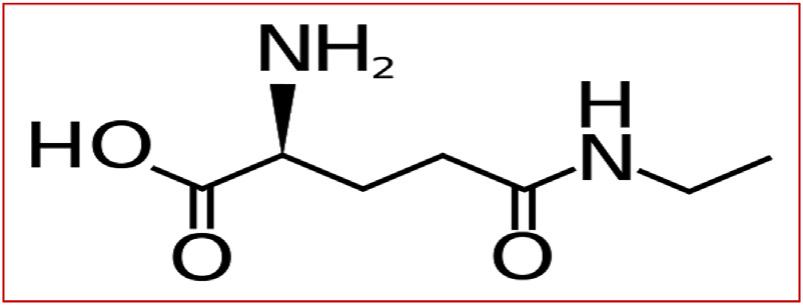
Chemical structure of L-theanine.

For synthetic L-theanine (Suntheanine), it is typically produced as racemic combination of L- and D-forms from food-borne ethylamine and L-glutamine by glutaminase enzyme as described by Juneja et al. (1999). The structural formula of L-theanine is shown in Figure 1.

The chemical structure of L-theanine is N-ethyl-L-glutamine; (2S)-2-ammonio-5-(ethylamino)-5-oxopentanoate, which was gained from the related proteinogenic L-AA glutamic acid (Scheid et al., 2012). The structural formula of L-theanine is shown in Figure 1.

## Pharmacokinetics of L-theanine

Animal studies have indicated that L-theanine absorption by the small intestine is facilitated by sodium-coupled active transporters (Kitaoka et al., 1996) and has been found that L-theanine was absorbed rapidly in the gastrointestinal tract (Van der Pijl et al., 2010). L-theanine, also called as Suntheanine, is known for its effect on alpha waves, which are produced in the brain of an individual during relaxed state. It was observed that 50 or 200 mg of L-theanine improved the alpha waves in occipital and parietal regions of the brain within 40 min after administration (Kakuda, 2002). The time to peak absorption after oral administration is 0.5 to 2 h in rats (Unno et al., 1999; Kakuda, 2002). The metabolism of L-theanine is even less clear. In 1966, ethylamine was detected in the urine of rats that were administered theanine, but the metabolic fate of theanine could not be determined (Tsuge et al., 2003). It is theorized that L-theanine undergoes hydrolysis in the kidneys and it is converted to glutamic acid (l-glutamate) and ethylamine by phosphate-independent glutaminase (Unno et al., 1999). Because theanine is metabolized by the kidneys, a large percentage of its byproducts are immediately excreted by the kidneys and not present in large quantities in the plasma (Tsuge et al., 2003). Theanine diffuses through the blood-brain barrier via the leucine-preferring transport mechanism (Yokogoshi et al., 1998; Juneja et al., 1999; Yokogoshi and Terashima, 2000). After dietary intake, L-theanine is absorbed through the small intestine and then hydrolyzed into ethylamine and glutamic acid in the intestine and liver followed by urinary excretion. Therefore, it is considered to function as a donor that supplies glutamic acid to the body (Tsuge et al., 2003). Aforementioned studies show that L-theanine has a strong anxiety effect, so it may be used as an antistressor agent for poultry industry.

## Beneficial effects of L-theanine

In previous studies, L-theanine has been reported as a functional ingredient with significant potential as food additive in designer foods (Vuong et al., 2011). Its health benefits have been associated with a number of pharmacological and biological properties such as antioxidant, growth promoter, immune booster, antistresser, hepatoprotective, antitumor, antiging, antimicrobial, anti-inflammatory, and antianxiety activities, observed in rats, nematodes, and *in vitro* studies (Saito et al., 2011; Gong et al., 2012; Culetu et al., 2016), and a summary of those available studies is presented in Table 1.

**Table 1:** Summary of the different study findings showing health-enhancing potential of tea amino acid L-theanine.

Agent	Species	Dosage	Study outcomes	Suggested/Observed effect	References
L-theanine	Mice	50, 100, 200 mg/kg	The tea amino acid theanine reduced the levels of liver enzymes (AST and ALT) and MDA, however, increased the activities of CAT, SOD, and GR	Antioxidant, hepatoprotective	Li et al., 2012
L-theanine	Mice	10 mg/kg	It reduced the levels of AST, ALT, and the expression of Bax and cleaved caspase-3.	Hepatoprotective	Nagai et al., 2015
L-theanine	Mice	50, 100, and 200 mg/kg	Tea amino acid theanine reduced the levels of bilirubin, AST, ALT, TNF-α, and IL-1β and also increased the activities of antioxidant enzymes and GSH contents.	Hepatoprotective, antioxidant	Jiang et al., 2012
L-theanine	Mice	10 and 100 mg/kg	L-theanine increased the contents of hepatic and heart glutamate without affecting in tumors, and also GSH.	Antioxidant, anticancer	Sugiyama and Sadzuka, 2004
L-theanine	Rats	8 mg/kg	Unique tea amino acid theanine reduced the different levels of aminotransferase, IL-1β, γ-GT, TGF-β, IL-6, and CTGF and the degree of lipid peroxidation.	Anti-inflammatory, hepatoprotective	Pérez-Vargas et al., 2016
L-theanine	Broiler	100, 200, and 300 mg/kg	It decreased the relative concentrations of cytokines IFN-γ and IL-2 in serum, and mRNA expression of IL-6 and TNF-α in thymus as well as IL-2 and IFN-γ in spleen. L-theanine also improved the antioxidative status by improving the serum GSH-Px, SOD, and relative CAT levels. In addition, it improved performance in broilers.	Immunomodulatory, antioxidant	Saeed et al., 2018
L-theanine	Broiler	100, 200, and 300 mg/kg	It has positive effects on immunity and gut microbe by increasing the community of commensal bacteria in poultry.	Antibacterial and immune modulator	Saeed et al., 2019
L-theanine	Broiler	400	It is a safe feed supplement up to 400 mg/kg and could boost the immune function of broilers.	Immunomodulatory	Wen et al., 2012
L-theanine	rats	4 g/kg (review)	It is found as promising liver protective agent via improving/restoring the antioxidant enzyme activities like SOD, CAT, and GSH, and inhibiting the production of MDA.	Antioxidant, hepatoprotective	Liang et al., 2015
L-theanine	Piglets	80 mg/kg	Tea amino acid (theanine) improved body weight gain, and anti-inflammatory cytokine production including TNF-α, IFN-γ, and IL-10.	Anti-inflammatory, growth promoter	Hwang et al., 2008
L-theanine	Poultry	Review	L-theanine amino acid as a powerful natural antistressor can also enhance productivity and health status in poultry industry.	Growth promoter, antistressor	Saeed et al., 2017
L-theanine	Human	10 or 100 mg/kg	L-theanine impeded the adriamycin (ADR) efflux from Ehrlich ascites carcinoma cells and sustained the concentration of ADR in cancer cells, however in normal tissue like liver and heart ADR increase was not noted.	Antitumor	Sadzuka et al., 1996
L-theanine	Mice	2 and 4 mg/kg	L-theanine significantly inactivated the NF-κB and ERK/p38, and prevent the oxidative damage of neuronal cells.	Memory enhancing	Kim et al., 2009

Abbreviations: ALT, alanine aminotransferase; AST, aspartate aminotransferase; CAT, catalase; CTGF, connective tissue growth factor; ERK, extracellular signal-regulated kinase; GR, glutathione reductase; GSH, glutathione; IL, interleukin; IFN, interferon; MDA, malondialdehyde; NF, nuclear factor; SOD, superoxide dismutase; TGF, transforming growth factor; TNF, tumor necrosis factor. TGF, transforming growth factor; ERK, extracellular signal-regulated kinase; CTGF,connective tissue growth factor; NF, nuclear factor;

### Lipid-Lowering Properties

Several experimental trials had proposed that L-theanine may have lipid-lowering properties. Zhang et al. (2002) studied the effect of theanine on hepatic tumor-induced hyperlipidemia in rats and found a significant decrease in all lipid indices. *In vitro* scientific evidences suggest that L-theanine may inhibit low-density lipoprotein peroxidation, thus decreasing the likelihood of developing atherosclerosis (Yokozawa and Dong, 1997). As such, the role of this AA (L-theanine) in lipid lowering may be similar to its proposed effects on blood pressure in mice; thus, theanine is a minor component contributing to an overall effect (Zheng et al., 2004).

### Antihypertension Effects

L-theanine AA of tea plant has proved to have antihypertensive effects in spontaneously hypertensive rats (Yokogoshi et al., 1995, 1998). Also in another study by Yokogoshi et al. (1995), they showed that high doses of L-theanine at 1,500 and 2,000 mg/kg resulted in significant decreases in blood pressure in spontaneously hypertensive rats, but glutamic acid and glutamine had no observable antihypertensive effects. In a clinical trial, 200 mg of L-theanine significantly reduced blood pressure increases in Japanese individuals during mental task (Yoto et al., 2012). A similar effect of L-theanine was observed in a double-blind randomized placebo-controlled study carried out in United Kingdom that showed L-theanine could antagonize the caffeine-induced blood pressure rise (Rogers et al., 2008). Although theanine may slightly reduce blood pressure, these studies suggested that other components in green tea might exert more of an antihypertensive effect.

### Immunomodulatory Effects

It is noticed in many studies that L-theanine and tea have favorable effects on immune function in animal models, cell cultures, epidemiological studies, and human interventions as well (Chattopadhyay et al., 2012; da Silva Pinto, 2013; Williams et al., 2019). In humans, γδ lymphocytes are a subset of T cells and considered as the first line of defense against many pathogens. The ethylamine is formed by the acid hydrolysis of L-theanine in the gastrointestinal tract and then via enzymatic breakdown mediated by the amidases in the liver, which are accomplished of expanding γδ T cells (Asatoor, 1966). Moreover, a previous clinical study recognized that the oral consumption of theanine enhanced γδ T-cell proliferation (Rowe et al., 2007). Therefore, this green tea AA that is known as L-theanine is being considered an essential compound for teas that have a strong ability to improve immune function. The immunomodulative actions of L-theanine are therefore very important for combating various infections and allergic diseases and hypersensitivity reactions. The results of another study have shown that the daily intragastric ingestion of theanine at 400 mg/kg could improve immune response through an increase in the splenic organ index and a decrease in the contents of interleukin-4/6/10 and CORT and the ratio of interleukin-4/interferon-γ in the serum of rats (Li et al., 2016).

### Increased Resistance Against Pathogenic Bacteria

**Figure 2:**
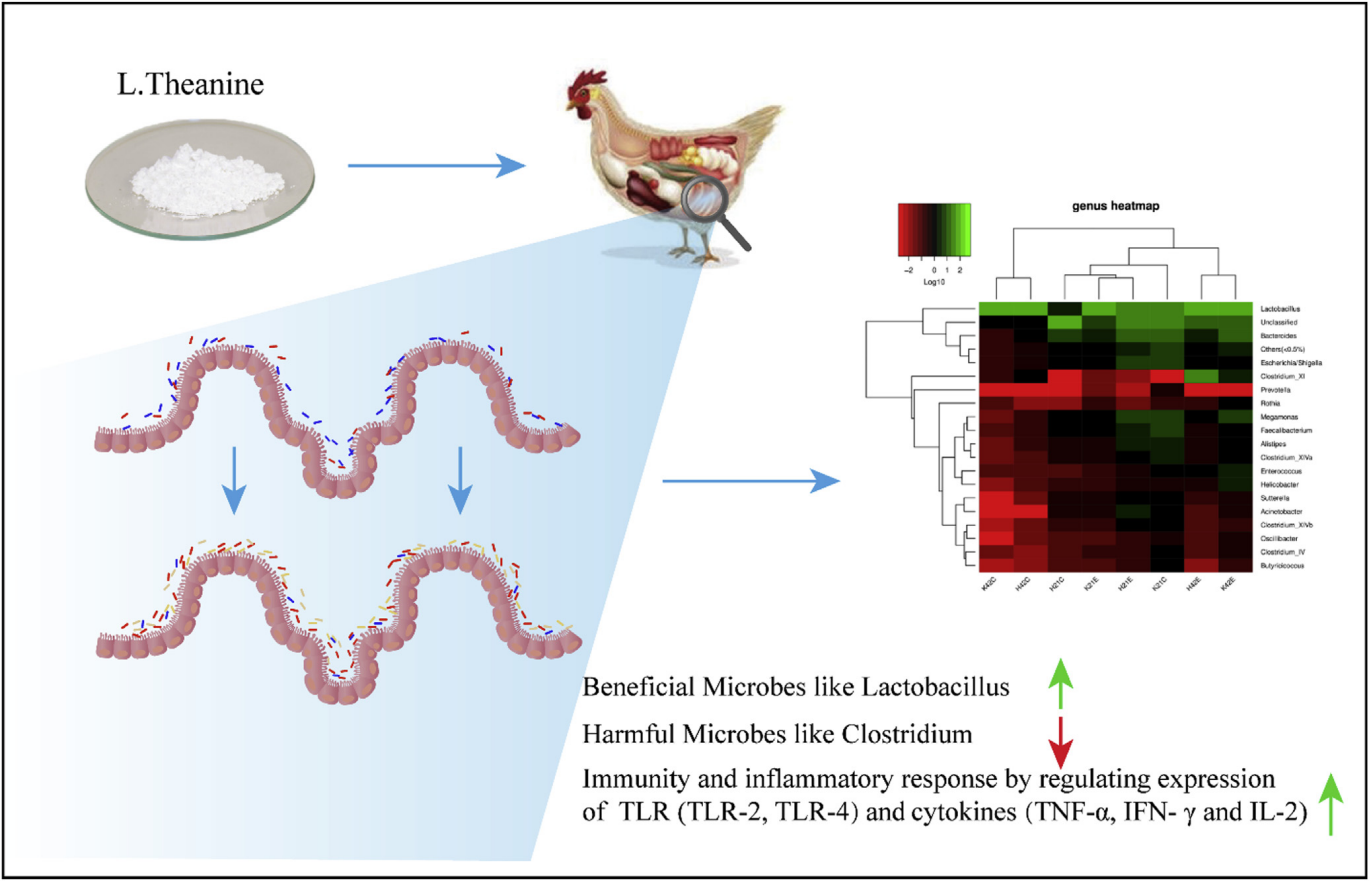
Beneficial effect of L-theanine on commensal bacteria. (Figure originally published in Saeed et al., 2019.)

It was assumed that drinking L-theanine–containing green tea could prime the immune system to react to bacterial infections by boosting the γδ T cells' infection-fighting abilities. This hypothesis was studied in 11 non–tea-drinking volunteers who drank about 1.3 mmol of theanine daily for either 2 or 4 wk (Kamath et al., 2003). In our previous study, we found that a diet with L-theanine revealed a potent positive impact on intestinal microbial communities by supporting useful bacteria such as *Lactobacillus* while decreasing harmful bacteria such as *Clostridium* as shown in Figure 2 (Saeed et al., 2019).

### Neuroprotective and Memory-Enhancement Properties

L-theanine offers a promising neuroprotective effect by binding to glutamate receptors and their receptor subtypes, that is, N-methyl-D-aspartate (**NMDA**), thereby preventing the excess calcium release into the extracellular space (Kakuda, 2002). Its ability to antagonize the NMDA receptor has gained peak gratitude because the introduction of memantine, an NMDA-receptor antagonist, indicated for the treatment of various diseases. L-theanine is also a more potent antagonist of α-amino-3-hydroxy-5-methyl-4-isoxazolepropionate and kainate receptors than NMDA receptors (Kakuda et al., 2002). The neuroprotective impact of L-theanine has been evaluated in many animal studies that primarily addressed its key pharmacologic effects (Kakuda et al., 2000). As described by Jamwal and Kumar (2017) who observed that when theanine was given to rats at 50 mg/kg/d, it exerts as neuroprotective effect as well as helps with preservation of striatal neurotransmitters homeostasis, antioxidant activity, anti-inflammatory potential, and free radical scavenging, which saves the neurons from death. L-theanine, at the rate of 2 to 4 mg/kg for 5 wk, notably attenuates the amyloid-induced memory impairment and neurons death. It was suggested that the beneficial effects of L-theanine on memory and learning capacities are due to the inactivation of extracellular signal–regulated kinase/p38 and nuclear factor kappa-light-chain-enhancer of activated B cells and decreased oxidative damage to neuronal cells (Deb et al., 2019).

### Antistressor Effect and Relaxation Properties

Green tea contains a large number of AAs including L-theanine, which represents around 50% of the total AAs (Horanni and Engelhardt, 2013). This AA (L-theanine) can protect the body cells from free radical species and prevent anxiety by increasing the levels of dopamine (**DA**) and serotonin in the nerve cells as well as helping to induce relaxation (Khan, 2014). Under heat condition, the supplementation of 200 mg/kg L-theanine and 200 mg/kg tea polyphenols in laying hen diets is the most appropriate technique for antioxidant impact. L-theanine remarkably improved CAT activity and decreased MDA in different organs and tissues (Wang and Cai, 2013). The alpha brain waves are being considered to be a measure of relaxation (Juneja et al., 1999). Their activity is associated to improved performance under stress, increased creativity, improved concentration and learning, and reduced anxiety (PDR Health, 2004). The effect of L-theanine on relaxation in humans was studied in 8 female college students suffering from low- and high-grade anxiety. It was observed that 200-mg dose of L-theanine significantly improved the alpha waves generation in the brains' parietal and occipital regions (Kobayashi et al., 1998). In the study of Takeda et al. (2012), they also reported that drinking water containing 0.3% L-theanine after birth markedly decreased the serum CORT concentration. Lee et al. (2010) stated that oral administration of L-theanine declines the levels of CORT (DA and noradrenaline), but increases 5-HT content in the brain cortex, striatum of mice, and hippocampus. Hence, L-theanine plays an imperative role to help in the secretion of neurotransmitters, hormones, and immune cytokines. Moreover, Tian et al. (2013) reported that L-theanine exerts a wide range of effects including natural antioxidant and neuroprotective effects against cognitive impairments in mice.

The results of White et al. (2016) encourage the antistress impact of L-theanine. Where, individual stress response to a cognitive stressor was notably decreased after 1 h of administration of L-theanine at 200 mg, and cortisol (CORT) response was outstandingly decreased 3 h after administration. In line with this, Kimura et al. (2007) found that individual stress and anxiety response to a cognitive stressor significantly reduced after receiving L-theanine (200 mg) compared to placebo. Also, a decrease in response of individual stress to a cognitive stressor was observed after administration of 200 mg of L-theanine (Yoto et al., 2012).

Research has demonstrated that long-term administration of L-theanine (400 mg daily) had antistress effects by reducing the salivary α-amylase and subjective stress (Unno et al., 2013). In addition, Steptoe et al. (2007) observed that administration of black tea for 6 wk decreased CORT levels and platelet activation in response to behavioral and cognitive stressors compared to control. The results of White et al. (2016) were consistent with some reports (Kimura et al., 2007; Yoto et al., 2012) of stress-reducing impact in response to an acute stressor, but others have stated treatment-related alterations in resting mood (Lu et al., 2004; Haskell et al., 2008). The discrepancy may be due to the variations in measurement instruments used and laboratory stressors; however, growing evidence supports a stress-reducing impact of L-theanine.

The fluctuation of blood levels of α1-acid glycoprotein and CORT could indicate physiological stress of poultry and animals. Regulation of stress was strongly correlated to the pituitary gland (hypothalamus) (Koolhaas et al., 1999; Dickerson and Kemeny, 2004). CORT is a glucocorticoid hormone, which plays an important role in mitigating stress. If animals were subjected to any external stimulation, the serum level of CORT would increase and metabolism of nutrients would be enhanced to cover the necessity of normal physiological functions (Nakamura et al., 1998; Liu et al., 2015).

L-theanine in broiler diets mitigated the elevated serum level of α1-acid glycoprotein on 25 d of age, concentration of IL-6 on 24 and 26 d of age, and the decreased the content of mucosal secretory immunoglobulin A in jejunum on 28 d of age of the lipopolysaccharide-challenged broilers (Li et al., 2018). Some reports indicated that L-theanine AA could enter through the blood-brain barrier to contribute in reducing responses of physiological and psychological stresses (Cho et al., 2008; Haskell et al., 2008; Yoto et al., 2012).

### Hepatoprotective and Antioxidant Effects

Hepatic injury is mostly caused by sustained exposure of the liver to harmful exogenous entities, including viruses, alcohol, toxicants, and also other biotransformed metabolites, which induced chronic inflammation of the liver, leading to diseases such as cirrhosis, fibrosis, and hepatocellular carcinoma.

To the best of our knowledge, tea is the only main dietary source of L-theanine. The most commonly consumed teas across the globe are nonfermented green tea and intensively fermented black tea (Bokuchava and Skobeleva, 1980). Nowadays, it has been proved that the green tea could provide protection against hepatic injury in toxic murine models including carbon tetrachloride, (Cui et al., 2014), dextran sodium sulfate (Inoue et al., 2013), microcystin (Xu et al., 2007), and alcohol (Park et al., 2012). In green tea, catechins are considered to be the most important functional components for liver protection. Compared with green tea, black tea exhibits a near complete destruction of catechins. However, black tea has also been shown to protect against carbon tetrachloride (Sun et al., 2013) and aflatoxin (Jha et al., 2013) and liver injury in mice. In general, theanine content seems to be minimally influenced by the fermentation process because both black and green teas contain similar theanine levels (Syu et al., 2008). Li et al. (2012) examined the effect of L-theanine on alcoholic liver injury both *in vivo* and *in vitro*. In ethanol-treated human hepatic cells, the L-theanine surprisingly protected hepatocytes against ethanol-induced cell cytotoxicity and apoptosis through the prevention of ethanol-triggered ROS and MDA. When mice were treated with ethanol, the level of hepatic enzymes such as alanine aminotransferase, aspartate aminotransferase, and MDA augmented as well as the activities of glutathione reductase, CAT, and SOD also diminished. However, when this AA (L-theanine) was administered to mice before ethanol exposure, all the baseline functions were restored. These results suggested that L-theanine may prevent ethanol-induced liver damage, possibly through enhanced hepatocyte antioxidative capacities (Li et al., 2012). Hence, it has been confirmed that the L-theanine is likely to be the compound responsible for hepatoprotection (Wang et al., 2017). The many possible beneficial effects of theanine consumption have been demonstrated by many animal studies and human trials. Regarding the possible mechanisms of actions of theanine on the downregulation of the inflammatory response, the induction of NO production and GSH synthesis are likely to be critical for the prevention of hepatic diseases as well as for the improvement of immune function as shown in the study by Wang et al., 2017.

## Usage of tea AA (L-theanine) in poultry

Poultry industry is providing the good-quality protein to human beings all over the globe. In many parts of the world, particularly in developing countries, poultry nutritionist are using antibiotics as growth promoters in poultry diet to improve the productive performance of birds; however, they are facing severe criticism by the consumers to replace synthetic antibiotics with safe natural alternatives (Ahossi et al., 2016; Saeed et al., 2017, 2018; Rehman et al., 2019). In a study on broiler chickens, Biswas and Wakita (2001) used 4 levels of *C. sinensis* powder (0.5, 0.75, 1.0, and 1.5%) in broiler diets and found that body weight gain and feed intake tended to decrease at a higher dose, while feed conversion ratio improved. Similarly, Uuganbayar (2004) claimed that 1.0 to 1.5% of green tea supplementation in broiler feed reduced body weight gain. Yang et al. (2003) conducted a trial to examine the effect of graded levels of green tea by-product on growth performance of broiler chickens; the results showed insignificant improvement in feed efficiency. Authors added that the inclusion of *C. sinensis* by-product to broiler diets decreased blood low-density lipoprotein cholesterol comparing to the control group and increased HDL and docosahexaenoic acid levels in blood. Moreover, cholesterol content in meat tended to decrease because of dietary inclusion of green tea by-product. In a most recent study, Zhang et al. (2020) reported the improved body weight gain, relative weight of intestine, intestinal histomorphology, antioxidant status, and intestinal mRNA levels of AA and peptide transporters while decreased the MDA content, crypt depth, jejunal protein carbonyls, and serum d-lactic acid by dietary administration of L-theanine in broilers. Similarly, in another study, 600 mg/kg supplementation of L-theanine in broilers ameliorated the effects of transport stress on meat quality by improving muscle pH, color, antioxidative status, and glycogen content while it decreased the drip loss and lactate and MDA contents of meat (Zhang et al., 2019a). A duck study used 300 to 1,500 mg/kg of L-theanine and reported 600 and 900 mg/kg as best supplementation levels to improve growth performance, immunity, intestinal morphology, and antioxidant status (Zhang et al., 2019b).

Also, Cao et al. (2005) demonstrated that feed consumption, feed conversion ratio, and body weight gain from 28 to 42 d of age were not enhanced, while mortality was expressively declined by supplemental *C. sinensis* by-products in broilers. Sarker et al. (2010) reported a statistical increase in body weight gain (1210.61 g/bird) in broiler chickens within the finishing period at the 0.5% level compared to the 1.0% (1033.36 g/bird) level of *C. sinensis* powder. Using a liquid hydroalcoholic extract of fresh *C. sinensis*, Erener et al. (2011) supplemented broiler diets with 0.1 g/kg or 0.2 g/kg extract of *C. sinensis* and reported the improved values of live body weight, feed conversion, dressing percentage, and carcass weight. The aforementioned authors attributed the improved growth performance with added green tea extract to physiological mechanisms such as the regulation of the microflora in caeca. On the other hand, the immune parameters (antibody level, serum lysozyme activity, intestinal secretory immunoglobulin, and serum interferon-γ and interleukin-2) of broilers were improved by the diets supplemented with L-theanine at 100, 200, 400, and 800 mg/kg diet (Wen et al., 2012).

**Figure 3:**
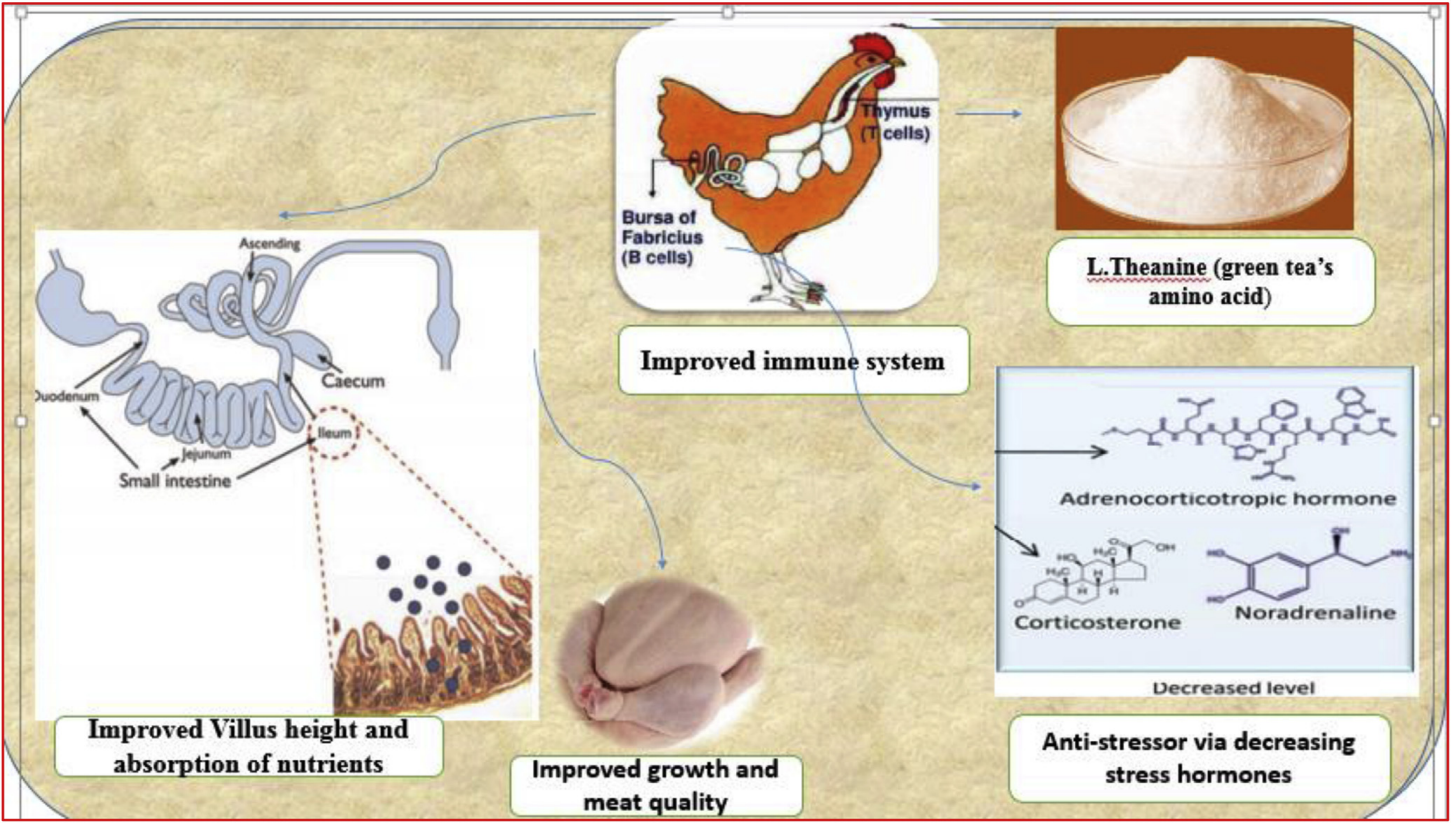
Flow diagram showing the proposed beneficial effects of L-theanine in poultry farming. (Figure originally published in Saeed et al., 2018.)

The live body weight, feed intake, and ileal digestibility of nutrients were not expressively affected by 10 g/kg supplementation of green tea in diet in comparison with control in broiler chickens. Plasma cholesterol and triglyceride were lowered with supplemental green tea compared to control birds (Afsharmanesh and Sadaghi, 2014). In the same context, El-Deek et al. (2012) concluded that dietary addition of green tea (1.5 and 3 g/kg diet) did not affect performance and meat quality of broiler. But, environmental emission and pollution via decreasing excreted nitrogen were overcome by the green tea treatments. Birds fed diets supplemented with graded concentrations of green tea extract (125, 250, 500, 1,000, and 2,000 mg/kg) had improved antioxidant and immunostimulant traits for broiler chickens (Farahat et al., 2016). Furthermore, the antibody titer against Newcastle disease virus vaccines was increased in chicks fed diets supplemented with green tea (Farahat et al., 2016). No adverse impacts on egg weight and egg production rate comparing to the control was observed when hens were fed diets containing 2.0% of green tea powder (Uuganbayar et al., 2005). Kojima and Yoshida (2008) did not find any important difference in egg production rate, egg mass, or egg weight between 1.0 and 0.0% green tea-supplemented laying hens. However, increasing the level of green tea up to 5.0 and 10.0% produced little effects in terms of egg production and egg contents. The same trend was observed by Biswas et al. (2000), who observed that long-term inclusion of layer diet with 0.60% Japanese green tea did not affect egg production or egg contents ratio. Ariana et al. (2011) postulated that enriching layer feeds with 1.50% powder of green tea and 0.50% extract of green tea did not significantly affect egg weight, feed intake, and egg production. On the other hand, Al-Harthi (2004) assured that dietary supplementation of green tea at 0.20% produced statistically improved egg weight and egg production comparing to the control group. In comparing the impacts of 2 levels (1 or 2%) of Japanese, Chinese, and Korean green tea on the performance of laying hens, it was found that egg yield of hens offered diets supplemented with 1 or 2% powders of green tea were statistically enhanced comparing to that of the control (Uuganbayar et al., 2006). Abdo et al. (2010) studied the influence of supplementing green tea extract (0.5–2.5 L/100 kg of diet) and leaves (1–5% of diet) in laying hen. The results showed significant improvements in feed conversion ratio, egg mass, and egg production due to supplementing the diet with 1% of green tea leaves compared to the control. With regard to egg quality criteria, Biswas and Wakita (2001) observed that using 0.3% of green tea powder as a dietary supplement improved the albumen percentage and Haugh unit score. The flow diagram indicating the possible beneficial effects of L-theanine in poultry in terms of growth promotion and stress reduction has been presented in Figure 3. By far, there is little work done on L-theanine into poultry, especially broiler chicken. There is a dire need to debate on the antistress effects of this AA to combat the stress problem of poultry industry.

## Toxicology effect of L-theanine

Regular or modest intake of green tea is safe (Boehm et al., 2009), but the ingestion of high doses (10–29 mg/kg/d) of green tea extract causes liver toxicity in humans (Lambert et al., 2007). The side effects of L-theanine in animal were not reported in any published study (Kakuda et al., 2000). It has been proposed that the side effects of all dietary additives, depend upon the sources, species, exposure duration and inclusion (Unl, 2012). As higher inclusion of L-theanine may lower blood pressure, appetite loss, difficulty concentrating, dizziness, diarrhea, headaches, nausea, and gastrointestinal discomfort (Haskell et al., 2008; Giesbrecht et al., 2010; Yoto et al., 2012). Chronic administration of L-theanine (250 mg/d for 8 wk) has also been found to be safe in patients with major depressive disorder, it even exhibited multiple beneficial effects against major depressive disorder symptoms including cognitive impairments, sleep disturbance, anxiety, and so on (Hidese et al., 2017). Similarly a 13-wk rodent study dealing with 1.5, 3, and 4 g/kg BW of L-theanine demonstrated no side effect on organ weight and gross pathology or histopathology (Borzelleca et al., 2006). In another study, maximum tolerable dose (0–5%) of L-theanine was added in the diet of rats that were analyzed for subacute (for 13 wk) and chronic (for 78 wk) toxicity. The results demonstrated zero side effects of L-theanine on feed intake, weight gain, or survival rate. Moreover, chronic administration (78 wk) significantly reduced the tumor number and tumor incidence without showing any toxicological effects (Fujii and Inai, 2008). However, there is no reported toxicological effect of L-theanine in in vivo and in vitro studies, and the Food and Drug Agency of the United States suggested that maximum consumption of L-theanine should not exceed 1,200 mg daily (Turkozu and Sanlier, 2017).

## Conclusion

L-theanine is a non–protein-derived AA that is abundant in leaves of *C. sinensis*. It could improve immune function by improving the spleen function and decreasing the CORT level in the serum. It acts as an antioxidant, growth promoter, immune booster, antistresser, hepatoprotective, antitumor, antiaging, antimicrobial, anti-inflammatory, and antianxiety agents. It could reduce the oxidative impairment via decreasing the lipid peroxidation, ROS production, and oxidative deterioration of macromolecules and increasing the glutathione concentrations. Furthermore, L-theanine might improve hepatocyte antioxidant efficiency via restoring the activities of antioxidant enzymes such as SOD, GSH, and CAT and inhibiting the formation of MDA in liver injury disorder. It has an attractive taste that can affect meat flavor and colors positively. To the best of our knowledge, most of earlier studies have been carried out in humans, pigs, and mice, but there is still a serious gap of information in literature on the use of L-theanine in farm animals and poultry, especially in broilers. So, this is the first comprehensive article on L-theanine with a new insight about the potential of this AA in poultry nutrition. We are hopeful that this article will be valuable for poultry scientist and nutritionist for potential use of L-theanine as a natural feed additive with potent antistressor functionality via decreasing the levels of CORT, DA, and noradrenaline and hepatoprotective properties. Future studies should be carried out to find its effective level that could be used on commercial level in poultry industry.
